# Brain tumor intelligent diagnosis based on Auto-Encoder and U-Net feature extraction

**DOI:** 10.1371/journal.pone.0315631

**Published:** 2025-03-24

**Authors:** Yaru Cao, Fengning Liang, Teng Zhao, Jinting Han, Yingchao Wang, Haowen Wu, Kexing Zhang, Huiwen Qiu, Yizhe Ding, Hong Zhu

**Affiliations:** 1 School of Medical Information and Engineering, Xuzhou Medical University, Xuzhou, Jiangsu, China; 2 Department of Computer Science and Engineering, State University of New York at Buffalo, Buffalo, New York, United States of America; Air University, PAKISTAN

## Abstract

Preoperative classification of brain tumors is critical to developing personalized treatment plans, however existing classification methods rely on manual intervention and often have problems with efficiency and accuracy, which may lead to misdiagnosis or delayed diagnosis in clinical practice and affect the therapeutic effect. We propose a fully automated approach to brain tumor magnetic resonance imaging (MRI) classification, consisted by a feature extractor based on the improved U-Net and a classifier based on convolutional recurrent neural network (CRNN). The encoder of the feature extractor based on dense block, is used to enhance feature propagation and reduce the number of parameters. The decoder uses residual block to reduce the weight of some features for improving the effect of MRI spatial sequence reconstruction, and avoid gradient disappearance. Skip connections between the encoder and the decoder effectively merge low-level features and high-level features. The extract feature sequence is input into the CRNN-based classifier for final classification. We assessed the performance of our method for grading glioma, glioma isocitrate dehydrogenase1 (IDH1) mutation status classification and pituitary tumor texture classification on two datasets, glioma or pituitary tumors collected in a local affiliated hospital and glioma imaging data from TCIA. Compared with commonly models and new models, our model achieves higher accuracy, with an accuracy of 90.72%, classified glioma IDH1 mutation status with an accuracy of 94.35%, and classified pituitary tumor texture with an accuracy of 94.64%.

## 1. Introduction

Brain tumors are considered among the deadliest and most difficult to treat of all forms of cancer [1]. Glioma is the most common type of brain tumor, accounting for 26.5% of brain tumors, and has a 20 - 30% of 5-year survival rate [2,3]. Pituitary tumor is another common type of brain tumor, accounting for about 16.2% of brain tumors, with evidence of increasing incidence [2]. Both of them impose great danger to people's lives and health. Previous studies indicate that to have effective personalized treatments for brain tumors, it is essential to determine the grade or category preoperatively [4,5]. The softness and density of pituitary tumors are related to many key issues, such as whether surgery is required, what kind of surgery to use, and what is the expected surgical effect. The preoperative grading of glioma has guiding significance for the development of surgical plan. Surgery is invasive, time-consuming, painful and useless for patients who are not suitable for surgery [6]. Such as diffuse midline glioma. Accurate and non-invasive preoperative glioma grading is essential for formulating treatment plans, implementing personalized treatment, prognosis, and predicting survival time [7–10]. In addition, the world health organization (WHO) redefines the classification of gliomas in the 2016 revision of central nervous system tumor classification. In addition to histological assessment, it also integrates molecular subtypes, such as isocitrate dehydrogenase (IDH). Molecular subtypes are mainly used for prognosis and postoperative targeted therapy. The prognosis of IDH mutated patients is often better than that of IDH wild-type patients. Therefore, preoperative grading and genetic mutation detection of glioma are very important, especially when tumor resection is not possible due to a high risk of severe postoperative complications and impairment.

However, the current preoperative classification or grading of pituitary tumors and gliomas usually use biopsy. Biopsy is an invasive procedure, and its potential risks outweigh the benefits. Biopsy may have an inherent sampling error. In addition, histopathological analysis and gene mutation detection are usually time-consuming and may delay diagnosis [11]. Therefore, an accurate non-invasive preoperative classification or grading method is very important.

The following are the major contributions of this paper:

(1) A feature extractor based on autoencoder is proposed for feature extraction of brain tumors. The autoencoder adopts improved U-Net frame and integrates Dense blocks and Residual blocks in order to get better features adaptively. The feature extractor is used for brain tumor feature extraction and image reconstruction. The more similar the input image and the output image, the more representative the extracted features.
(2) A classifier based on convolutional recurrent neural network (CRNN) is proposed. The feature sequences extracted by the feature extractor are input into CRNN for brain tumor grading or classification.
(3) The model uses 3D image sequences instead of 2D (slice) classification methods, and does not require pre-segmentation of brain tumors or manual extraction of a large number of features.

This paper proposes a brain tumor intelligent diagnosis model based on autoencoder optimized feature extraction to assist the clinical diagnosis of gliomas and pituitary tumors and help formulate follow-up treatment plans.

The rest of the paper is organized according to the following pattern. Section 2 gives the related works. Section 3 describes the data set used in this paper. Section 4 presents the proposed methodology for improved U-Net-based feature extractor, CRNN-based classifier, and brain tumor intelligent diagnosis model composed of them. Section 5 discusses the results of the experiments. Section 6 provides discussion of current work and Section 7 is devoted to conclusions.

## 2. Related work

With the rapid development of medical imaging technology, some non-invasive Radiomics-based methods for diagnosing glioma or pituitary tumors are emerging. Wang et al. [12] adopted fractional anisotropy and apparent diffusion coefficient (ADC) parameters, obtained from the diffusion tensor imaging, to differentiate grade II and grade III gliomas. They concluded that minimum ADC values can lead to better diagnostic performance in differentiating grades II and III gliomas, and the predictive diagnostic equation could be useful for the differentiation. Peng et al. [13] used radiomics features from multiparameter magnetic resonance imaging (MRI) to identify IDH genotypes of glioma. They extracted a total of 851 radiomics features on each volume of interest (VOI) of three sequences, including contrast-enhanced-T1 weighted (CE-T1 W), T2 weighted (T2 W) and arterial spin labeling (ASL). All radiomics features were processed by the Pearson test. Finally, the accuracy of the classifier, which combines the features of all three sequences, achieved 0.823. Chen [14] judges the softness level of pituitary tumor by looking for the relationship between the signal level of MRI T1 W, T2 W and pituitary tumor texture. He concluded that the lower the signal displayed on T2 W, the more likely pituitary tumor texture hard. However, methods based on Radiomics usually require people to delineate the region of interest and then extract a large number of features, which are slightly lacking in efficiency and accuracy.

With the popularization of artificial intelligence, medical image processing methods have gradually changed. Shallow machine learning and deep learning methods are increasingly used in medical image processing [15]. Sudre et al. [16] combined MRI techniques with machine learning methods. They extracted the shape, intensity distribution (histogram), and rotation invariant Haralick texture features over the tumor mask, and then used the extracted features to predict grades using a random forest algorithm. In the end, gliomas were correctly stratified by grade in 53% of the cases (87% of the gliomas grades predicted with distance less than 1). Im et al. [17] used deep learning methods for glioma grading using whole-slide images obtained from routine clinical practice. They trained a deep transfer learning method using the ResNet50V2 model to classify grades of diffuse gliomas. The accuracy of the diffuse glioma grading model was 0.68. Zhang et al. [18] used the clinical features of multimodal MRI combined with random forest machine learning algorithms to predict IDH mutation status with 86% accuracy. These methods generally require manual pre-segmentation of tumors and use 2D (slice) classification methods. Choi et al. [19] used a 3D U-shaped model for glioma segmentation, and then selected 5 images with the largest tumor for each patient based on the segmentation results as the image input of the second model (34-layer Resnet) to predict glioma IDH1 Mutation status. They finally achieved an accuracy of 0.787 on the cancer imaging archive (TCIA) data set. Khan et al. [20] proposed a hierarchical deep learning-based brain tumor classifier using convolutional neural network (CNN). The model classified the input into four classes, glioma, meningioma, pituitary, and no-tumor, and accomplished 92.13% accuracy. Akter et al. [21] proposed a deep CNN-based architecture for automatic brain image classification into four classes and a U-Net-based segmentation model, and achieved the highest accuracy of 98.7% in a merged dataset and 98.8% with the segmentation approach. Qureshi et al. [22] specifically designed RobU-Net based modified U-Net for handling Rician noise in MRI scans, to improve the accuracy and robustness of MRI image segmentation. Jia et al. [23] applied structural, morphological, and relaxometry detailsa into a fully automatic heterogeneous segmentation using support vector machine, which achieved almost 98.51% accuracy.

Image feature extraction is very important in medical image processing tasks. Effective feature extraction can significantly improve the performance of a model because it helps the model capture key information and patterns in the image. Qureshi et al. [24] proposed a novel two-stage MGMT promoter methylation prediction system by relating the genomic variation with radiomics features. Saba et al. [25] applied the Grab cut method for accurate segmentation of actual lesion symptoms, then concatenated hand crafted features with features from fine-tuned visual geometry group. Faruqui et al. [26] proposed LungNet consists of 22-layers CNN, which combines latent features that are learned from CT scan images and MIoT data to enhance the diagnostic accuracy of lung classification. Aurna et al. [27] built the two-stage ensemble model by pre-trained the best models and concatenated in two stages for feature extraction, and used principal component analysis to select most substantial features.

Autoencoder is an unsupervised model in deep learning which can automatically extract many deep features from the medical image data sets. Myronenko [28] built a segmentation network for tumor subregion segmentation from 3D MRIs based on encoder-decoder architecture. He added the variational autoencoder branch to the network. The encoder is used to extract image features, and the decoder and branch are used to reconstruct the segmentation mask and the original image, respectively. Denner et al. [29] used an autoencoder-based structure to segment multiple sclerosis lesions in longitudinal brain MR scans. Xu et al. [30] developed a network called cxnet-m1 to help diagnose abnormalities in chest X-ray images. Fan et al. [31] relied on quadratic artificial neurons to build an encoder-decoder structure called quadratic autoencoder, and applied it to denoise low-dose computed tomography images. Gu et al. [32] introduced a context encoder network for segmenting 2D medical images. Their method has shown good performance in segmenting the images of disc, vessel, lung, cell contour, and retinal optical coherence tomography layer. These examples all demonstrate that autoencoder has great potential in solving many problems in medical image processing. The autoencoder has demonstrated good utility and robustness in image denoising and model efficiency. The autoencoder approach can better cluster/group the features of the encoder endpoints.

Although the application of autoencoders in medical image processing has demonstrated its strong potential and flexibility, there are still some shortcomings and challenges. First, autoencoder models, especially deep autoencoders, usually have a large number of parameters. These parameters need to be iteratively updated by backpropagation algorithms to minimize reconstruction errors. Due to the high resolution and complexity of medical image data, training autoencoder models often takes a long time. Second, when processing highly complex or diverse medical images, autoencoders may not be able to fully capture all the important features in the image, and if there is significant variability or abnormality in the image in the data set, the autoencoder may miss some key details.

Therefore, an autoencoder-based method for brain tumor feature extraction is proposed, which uses an improved U-Net frame and combines Dense block to reduce the number of parameters while enhancing feature reuse and propagation of valid features and Residual block to avoid the disappearance of gradients. We use a CRNN-based classifier to classify the feature sequences to classify brain tumors.

## 3. Data and materials

The datasets used in our studies were preoperative images of glioma or pituitary tumors collected in a local affiliated hospital from January 23, 2018 to March 13, 2023, and we accessed these date on June 6, 2023. All these data are the two modalities of T1-MRI and T2-MRI image data collected with four 3.0-Tesla instruments before surgery. The MRI parameters of glioma are shown below. TI-MRI: repetition time (TR) is 2140.05ms, echo time (TE) is 15.488ms, flip angle (FA) is 90°, and resolution is 0.4688mm. T2-MRI: TR is 4300ms, TE is 121.4464ms, FA is 90°, and resolution is 0.4688mm. The MRI parameters of pituitary tumors are shown below. TI-MRI: TR is 360ms, TE is 17ms, FA is 90°, and resolution is 0.3906mm. T2-MRI: TR is 3000ms, TE is 131.248ms, FA is 90°, and resolution is 0.3516mm. T1 W MRI was performed with gadolinium. Each patient had MRI data of transverse plane (OAX), sagittal plane (OSAG) and coronal plane (OCOR). In this experiment, we used patient image sequences with OAX orientation for glioma, and we used patient image sequences with OCOR orientation for pituitary tumor. Image sequence refers to a series of images that are successively acquired on the target at different times. The MRI parameters of glioma and pituitary tumor are shown in Table 1. WHO classifies gliomas into grades I to IV according to histological morphology. Grades I and II are low-grade gliomas, and grades III and IV are high-grade gliomas [33]. IDH mutation status is divided into IDH-wildtype and IDH-mutant. Pituitary tumors are divided into soft texture and hard texture according to their softness level.

**Table 1:** The MRI parameters of glioma and pituitary tumor.

Tumor type	MRI sequence	TR (ms)	TE (ms)	FA (°)	Resolution (mm)	MRI Plane
Glioma	T1	2140.05	15.488	90	0.4688	OAX
	T2	4300	121.4464	90	0.4688	
Pituitary tumor	T1	360	17	90	0.3906	OCCR
	T2	3000	131.248	90	0.3516	

Glioma patients range from 8 to 77 years old, with an average age of 53.7 ± 11.7; Pituitary tumor patients range from 13 to 73 years old, with an average age of 50.4 ± 12. These brain tumor images were all labeled by professional clinicians based on the results of brain tumor biopsy. Each image of glioma has a grading label (low-grade or high-grade) and an IDH1 mutation status label (IDH1-wildtype or IDH1-mutant). The final glioma dataset includes a total of 4896 MRI images of 153 data items (78 low-grade, 75 high-grade and 95 IDH1-wildtype, 58 IDH1-mutant). The final pituitary tumor dataset includes a total of 3288 MRI images of 137 data items (90 soft texture, 47 hard texture). In addition, the study also collected 153 cases of glioma imaging data from TCIA (72 low-grade, 81 high-grade and 95 IDH1-wildtype, 58 IDH1-mutant), and the corresponding label came from TCGA. The study used methods such as reflection and rotation to perform data augmentation on the glioma and pituitary tumor imaging datasets. Two sequences (i.e., T1 and T2) were applied in the study. Each sequence of glioma has 16 slices and each sequence of pituitary tumor has 12 slices. T1 and T2 images were registered to an identical 1-mm isovoxel spatial coordinate. The images of glioma were subjected to signal intensity normalization and resampling to sizes of 256 ×  256 ×  32, and the images of pituitary tumor were subjected to signal intensity normalization and resampling to sizes of 256 ×  256 ×  24. The data sets for glioma and pituitary tumors are shown in Table 2.

**Table 2:** The data sets for glioma and pituitary tumors.

Tumor type	Age range	Average age	Data item			Slice
Glioma	8 ~ 77	53.7 ± 11.7	Low-grade	78	153	4896
			High-grade	75		
			IDH1-wildtype	95	153	4896
			IDH1-mutant	58		
Pituitary tumor	13 ~ 73	50.4 ± 12	Soft texture	90	137	3288
			Hard texture	47		

**Fig 1:**
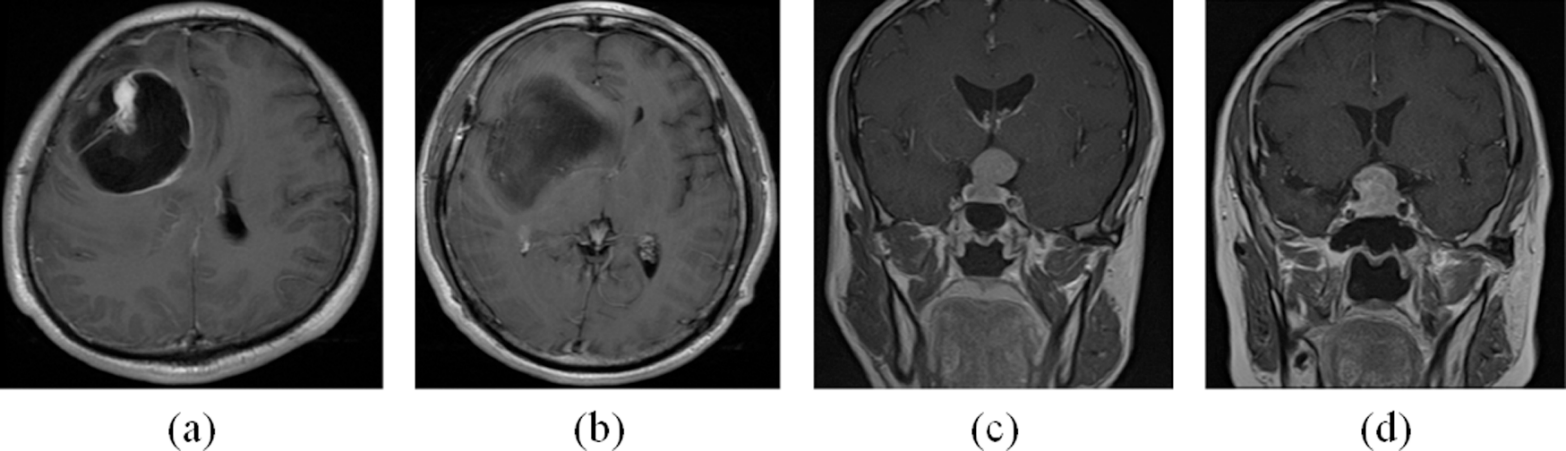
Gliomas and pituitary tumors. (a) High-grade glioma with IDH1-wildtype. (b) Low-grade glioma with IDH1-mutant. (c) Pituitary tumor with soft texture. (d) Pituitary tumor with hard texture.

Each image of pituitary tumor has a category label from the following two categories: soft texture and hard texture. Fig 1a and 1b show gliomas of different grades and different IDH1 mutation states. Fig 1c and 1d are pituitary tumors of different textures.

## 4. Brain tumor intelligent diagnosis method based on U-Net feature extraction

Our proposed brain tumor intelligent diagnosis model consists of three parts: (1) the feature encoder module; (2) the feature decoder module; (3) classification module based on CRNN.

### 4.1. U-Net

U-Net [34] is a symmetrical U-shaped network, with a contracting path on the left side and an expansive path on the right side. The contracting path is composed of convolutional layers, which are used to extract image features to obtain feature maps. The expansive path consists of upsampling layers to restore the extracted feature maps. The feature maps obtained from each convolutional layer on the left in the U-Net network will be concatenated to the corresponding upsampling layer using skip-connection. This enables the U-Net to realize the fusion of high-level features and low-level features, avoid information loss to a certain extent, and improve the accuracy of the model. U-Net network is often used for medical image segmentation. It is widely improved and used for its excellent feature extraction ability [35–37]. Therefore, we can make use of the nice properties of the U-Net network and its excellent feature extraction ability to design a U-Net-based feature extractor for glioma sequence images and pituitary tumor sequence images.

### 4.2. Encoder based on Dense block

The contracting path of U-Net is a particularly critical component of the network architecture, responsible for gradually reducing the spatial dimension of the input image while gradually increasing the number of channels of the feature map to extract higher-level, more abstract image features. To further enhance the feature extraction capability of the U-Net model, we used a Dense block to replace the original block in the contracting path.

Dense block is a structure with dense interlayer output, which can significantly improve the expressiveness of the model and the reuse rate of features. In traditional convolutional networks, the input of each layer only comes from the previous layer, while Dense blocks take the output of all previous layers as the input of subsequent layers, making information flow and share more fully in the network.

**Fig 2:**
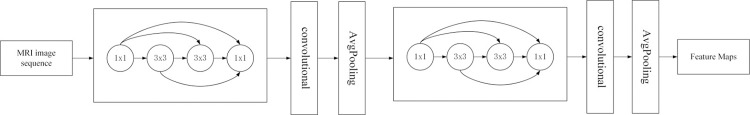
Contracting path based on a Dense block.

As shown in Fig 2, the entire contracting path uses two Dense blocks. Each Dense block consists of 4 convolutional layers with 64, 64, 128, and 128 kernels, respectively. The kernel size in each convolution layer is 1 * 1, 3 * 3, 3 * 3, 1 * 1. The 1 * 1 convolution is used to reduce and integrate features, and the 3 * 3 convolution is used to capture local spatial features, which ensures that the model can extract rich features at different receptive field scales. Through this hierarchical convolutional design, the detailed features in the image can be captured better, while the number of parameters is reduced, and the computational efficiency of the network is improved. The input of each convolutional layer is the sum of the outputs of all the previous layers, and each convolutional layer uses LeakyReLU as the activation function. The two blocks are connected by a 1 * 1 convolution layer to further reduce the number of channels, and downsampled using a 2 * 2 AvgPooling layer. The process of downsampling gradually focuses on more recognizable features by reducing spatial resolution, while also reducing the amount of computation.

The use of Dense block can help us reduce the number of parameters while enhancing feature reuse to strengthen the spread of effective features. We input the glioma image sequence or pituitary tumor image sequence into such a contracting path for encoding and obtain the feature map sequence retaining rich spatial and contextual information.

### 4.3. Adaptive optimization of feature extraction based on U-Net

The main function of the expansive path is to gradually restore the spatial resolution of the image, and at the same time combine the low-level features and high-level semantic information to reconstruct the accurate output image. In this path, it is very important to keep the integrity of feature transmission and avoid the loss of feature information. To further optimize feature extraction, we replace the original convolutional blocks with a Residual block in the extended path.

The Residual block is an effective structure to solve the gradient disappearance problem in deep networks by introducing skip connections, which allows information to pass directly by bypassing the intermediate convolutional layer by connecting the input directly to the output. This structure is especially useful in deep networks, ensuring that gradients can propagate more efficiently, thus avoiding training difficulties caused by increased depth.

**Fig 3:**
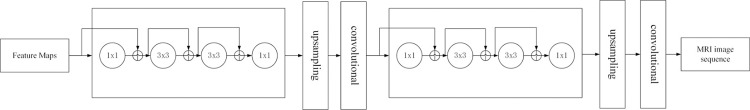
Expansive path based on Residual block.

As shown in Fig 3, the entire expansive path has two Residual blocks. Each Residual block uses 4 convolutional layers with 64, 64, 128, and 128 kernels, respectively. The kernel size in each convolution layer is 1 * 1, 3 * 3, 3 * 3, 1 * 1. Each convolutional layer uses the LeakyReLU as the activation function. In order to enhance the function of residual blocks, we add a shortcut connection at the top of each block to directly add the input of the block to the output of the block, so as to realize the direct connection between layers, better maintain the gradient flow of the network, avoid the gradient disappearing, and thus improve the learning ability of the model.

We relied on the upsampling layer to increase the dimensions of the feature map and then concatenate it to the feature map of the symmetric contracting path. This enables us to avoid the loss of low-level features. The use of the Residual block can effectively deepen the depth of the U-Net and meanwhile avoid the vanishing of the gradient, which leads to better image reconstruction. In the end, we obtained an image sequence with the same dimensions as the original input one.

We compared the corresponding pixels of the generated image and the original one, then updated the model weight, and reduced the loss through backpropagation. The lower the loss, the more similar the generated image and the original one. The generated image was obtained by decoding the features extracted by the encoder. Therefore, the more similar the final generated image and the original one, the more representative the extracted features are. After extracting the required features, we can perform subsequent grading or classification operation.

### 4.4. A multi-sequence brain tumor grading or classification model

**Fig 4:**
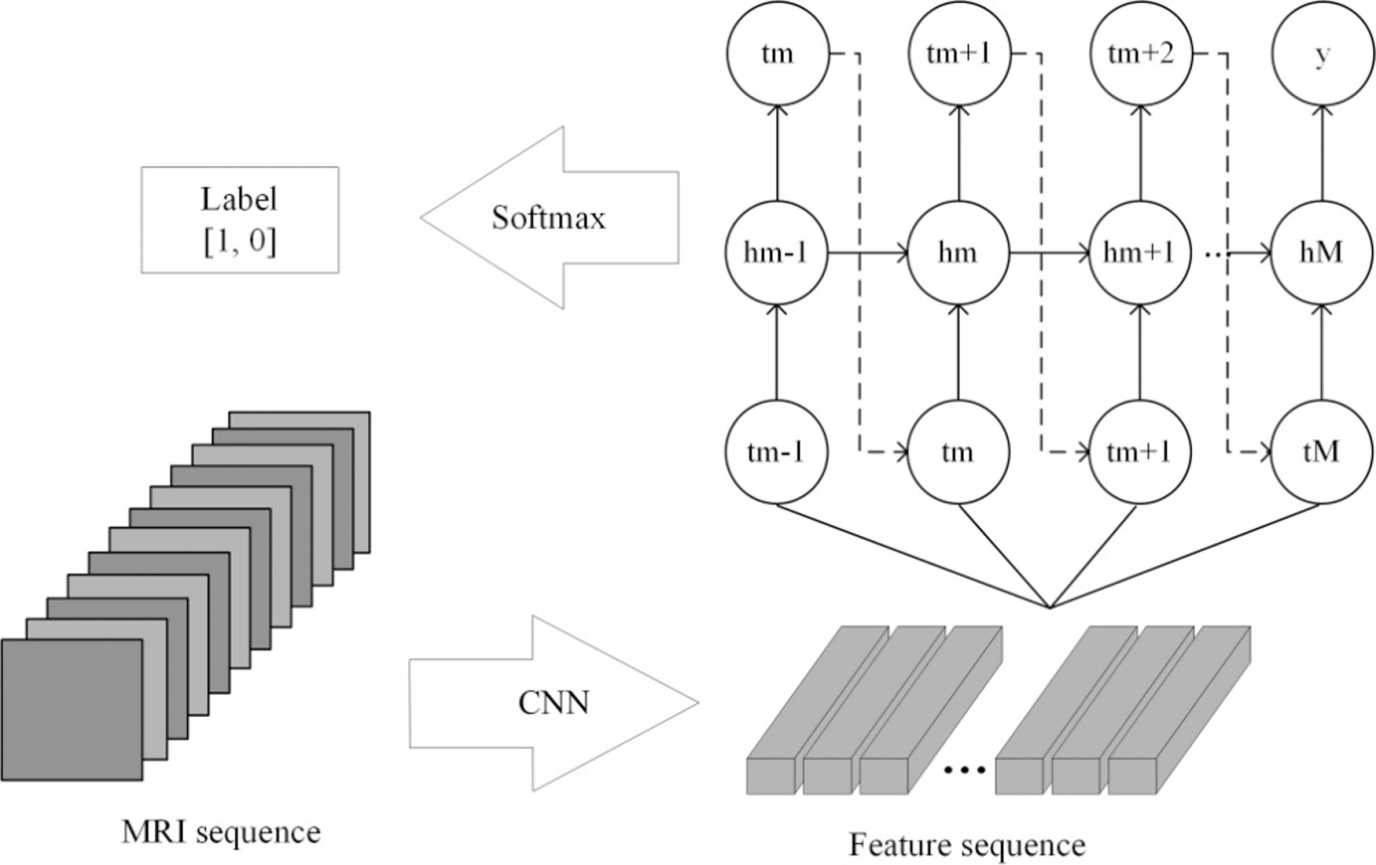
The architecture of CRNN.

CRNN [38] consists of a CNN and a recurrent neural network (RNN), which is capable of processing sequence data. Fig 4 shows the architecture of CRNN. Firstly, initial feature extraction is carried out on the input data through the CNN layer, and the spatial features of the input image are extracted step by step. These features can include local image information such as edge, texture and shape. With the deepening of the network, CNN can gradually extract more abstract and high-level features from lower-level features. These CNN-processed features are not used directly for the final classification or regression task, but are passed as input to the RNN section. RNN is a model that can capture sequence information and is good at dealing with time-dependent tasks. RNN remembers and passes the information of the previous time through its loop structure, so that it retains the timing relationship and context information in the sequence when processing sequence data.

The MRI images of brain tumors are image sequences corresponding to a single label. This means that the MRI image data of each glioma patient or pituitary tumor patient can be viewed as a sequence data. Therefore, we can input the features extracted from the U-Net-based feature extractor into CRNN for final grading or classification. As mentioned earlier, the brain tumors images of each patient include two sequences, T1 and T2. In order to achieve better classification results, we merged the two sequences to form a multi-modality sequence and then performed the subsequent feature extraction and classification on it.

**Fig 5:**
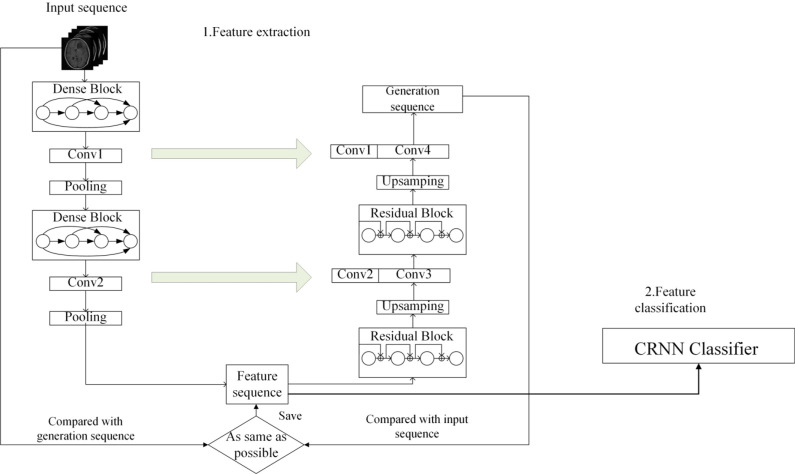
Brain tumor intelligent diagnosis model.

As shown in Fig 5, we input glioma image sequence or pituitary tumor image sequence into the U-Net-based feature extractor. The shrink path extracts advanced features from the image based on Denseblock, encodes the image sequence layer by layer, and generates a series of feature maps. The expansion path decodes the encoded feature map based on Residual block and restores to the same spatial resolution as the original input image. At the same time, low level spatial information and high level semantic information are combined to generate a new image sequence. We compare the generated image sequence with the original input image sequence to find the most representative feature map sequence.

After completing the feature extraction, we used the extracted feature map sequence as the input to CRNN for the final grading or classification of the brain tumor images. The CNN in CRNN conducts further feature extraction on the input feature sequence, including more complex pattern and structural information. The RNN captures the time dependence and the relationship between sequences in the image sequences through the cyclic structure, and performed a feature sequence prediction on this basis. Finally, CRNN converts the processed feature mapping sequence into a tag sequence through the transcription layer, which is the final classification result. The entire model is suitable for feature extraction and classification of image sequences, while avoiding the problems of too many parameters and high computational cost in 3D models [39].

## 5. Experimental results

### 5.1. Experiment platform

We conducted our experiments on a machine that runs Windows 10 operating system and is equipped with a 2.10GHz Intel Xeon (dual core) processor, 64 GB memory, and a GeForce RTX 2080Ti graphics card. We used PyCharm as the development environment, Keras as the deep learning framework, and Python as the programming language.

### 5.2. Brain tumors grading or classification based on U-Net feature extraction

Before classifying the brain tumor images, we first used the U-Net-based feature extractor to extract features from the brain tumor image sequences. We fed the glioma images or the pituitary tumor images to the U-Net-based feature extractor to train and extract the required features.

**Fig 6:**
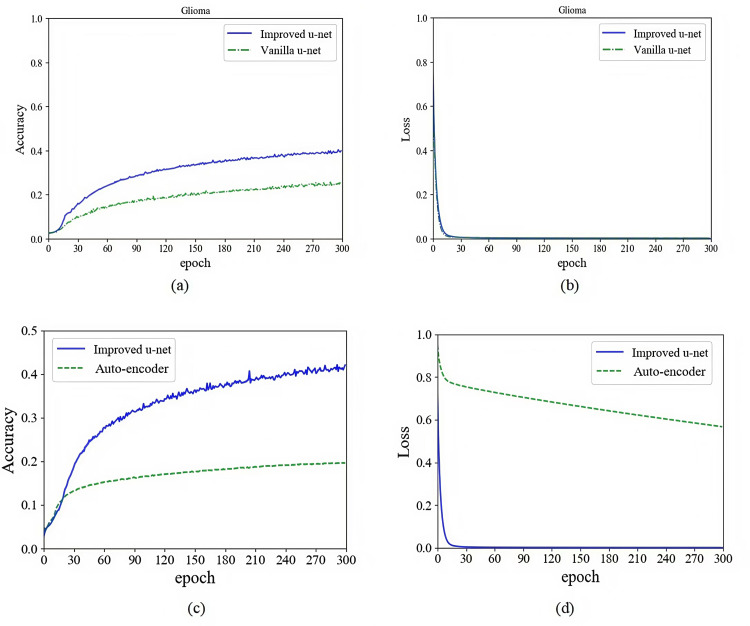
Performance comparison of autoencoder, vanilla U-Net and improved U-Net in glioma feature extraction. (a) Comparison of vanilla U-Net and improved U-Net in feature extraction accuracy. (b) Comparison of vanilla U-Net and improved U-Net in feature extraction loss. (c) Comparison of autoencoder and improved U-Net in feature extraction accuracy. (d) Comparison of autoencoder and improved U-Net in feature extraction loss.

**Fig 7:**
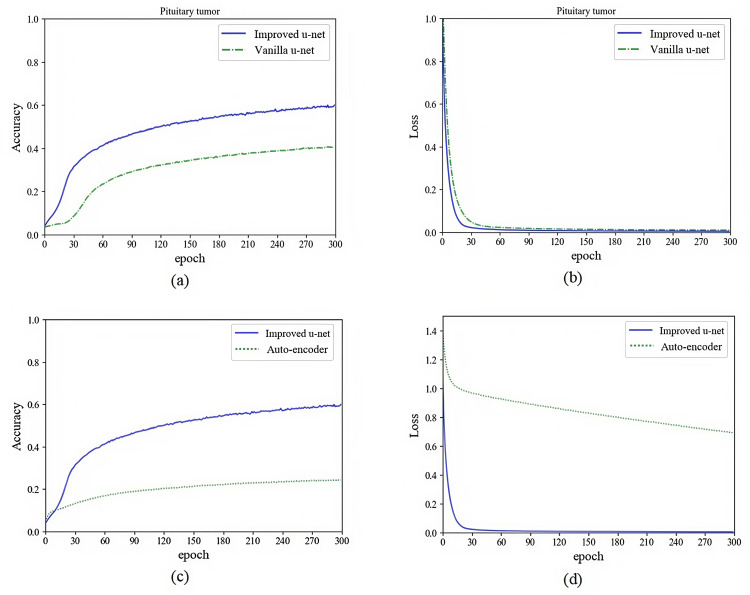
Performance comparison of autoencoder, vanilla U-Net and improved U-Net in pituitary tumor feature extraction. (a) Comparison of vanilla U-Net and improved U-Net in feature extraction accuracy. (b) Comparison of vanilla U-Net and improved U-Net in feature extraction loss. (c) Comparison of autoencoder and improved U-Net in feature extraction accuracy. (d) Comparison of autoencoder and improved U-Net in feature extraction loss.

In order to prove the effectiveness of the method proposed in this study, we used multi-sequences of glioma and pituitary tumor images to train models based on autoencoder, vanilla U-Net and improved U-Net, and compared their training processes. Networks were implemented using an adaptive moment estimation (Adam) optimizer and a mean square error (MSE) loss function. The initial learning rate was set to 10^ −5^ with a batch size of 4. We stopped training the models when the loss curve reached its lowest point. The specific training processes and comparison are shown in Figs 6 and 7.

**Fig 8:**
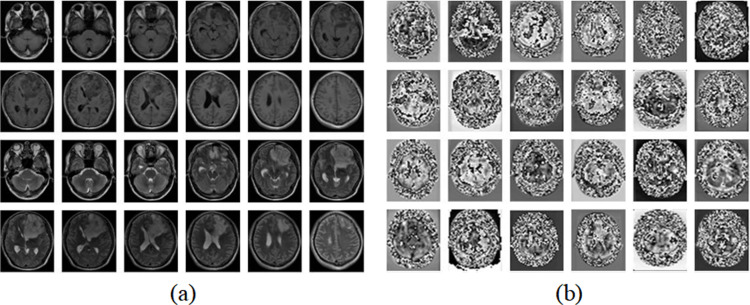
Example of glioma feature extraction. (a) The input image sequence. (b) The corresponding feature map sequence.

**Fig 9:**
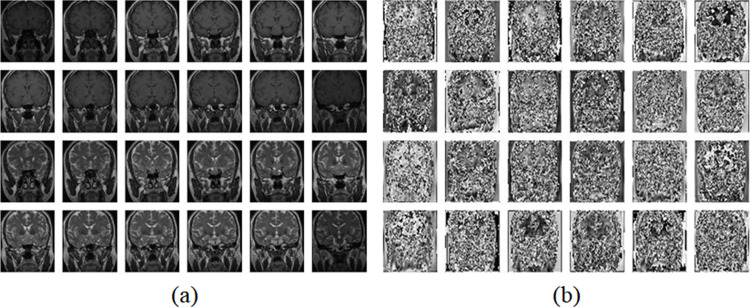
Example of pituitary tumor feature extraction. (a) The input image sequence. (b) The corresponding feature map sequence.

As shown in the figures above, we trained the models for 300 epochs. It can be seen from the figures that the accuracy of using improved U-Net to extract features is significantly higher than that of autoencoder and vanilla U-Net, and the convergence speed of loss using improved U-Net is much faster than that of autoencoder and vanilla U-Net. As shown in Figs 8 and 9 are the input sample image sequence and its corresponding feature map sequence extracted by the improved U-Net feature extractor.

For further comparison, we input the features extracted by the three models (autoencoder, vanilla U-Net and improved U-Net) into the CRNN classifier for final grading or classification. CRNN classifier was implemented using an Adam and a cross entropy (CE) loss function. The initial learning rate was set to 10^ − 4^ with a batch size of 10. In addition, in order to avoid over-fitting, this experiment used dropout in the CRNN classifier. The dropout ratio is set to 0.5. To achieve better results, we have conducted the grading or classification training and testing on the three sequence data, including multi-sequence, T1 and T2 of glioma images and pituitary tumor images.

**Fig 10:**
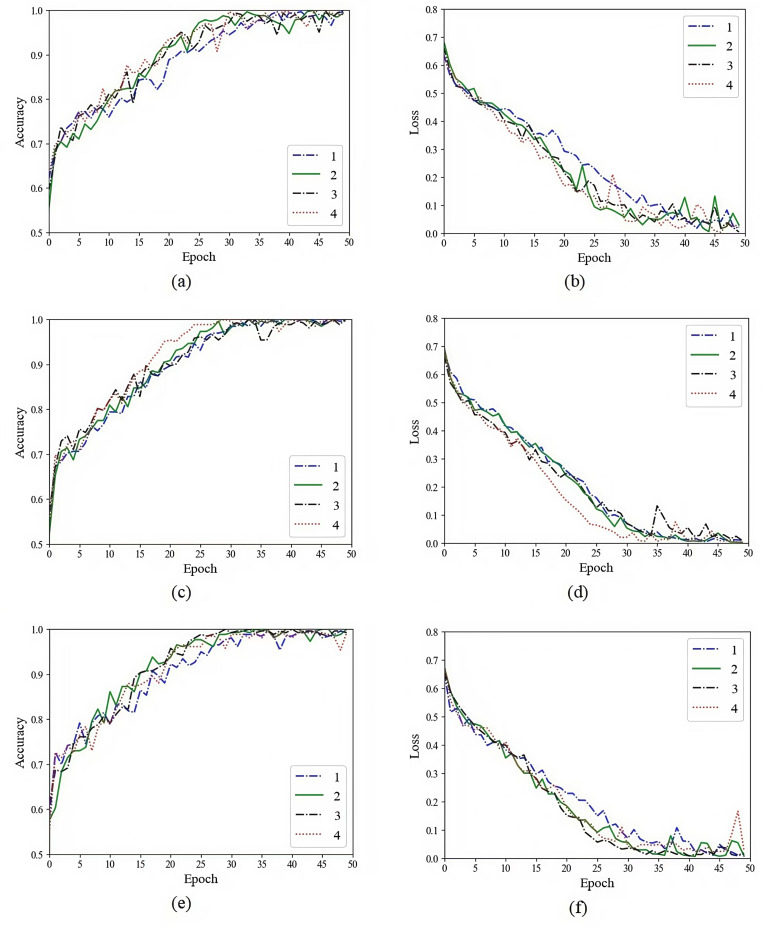
The glioma grading training process of the improved U-Net model. (a) Multi-sequence training accuracy. (b) Multi-sequence training loss. (c) T1 training accuracy. (d) T1 training loss. (e) T2 training accuracy. (f) T2 training loss.

**Fig 11:**
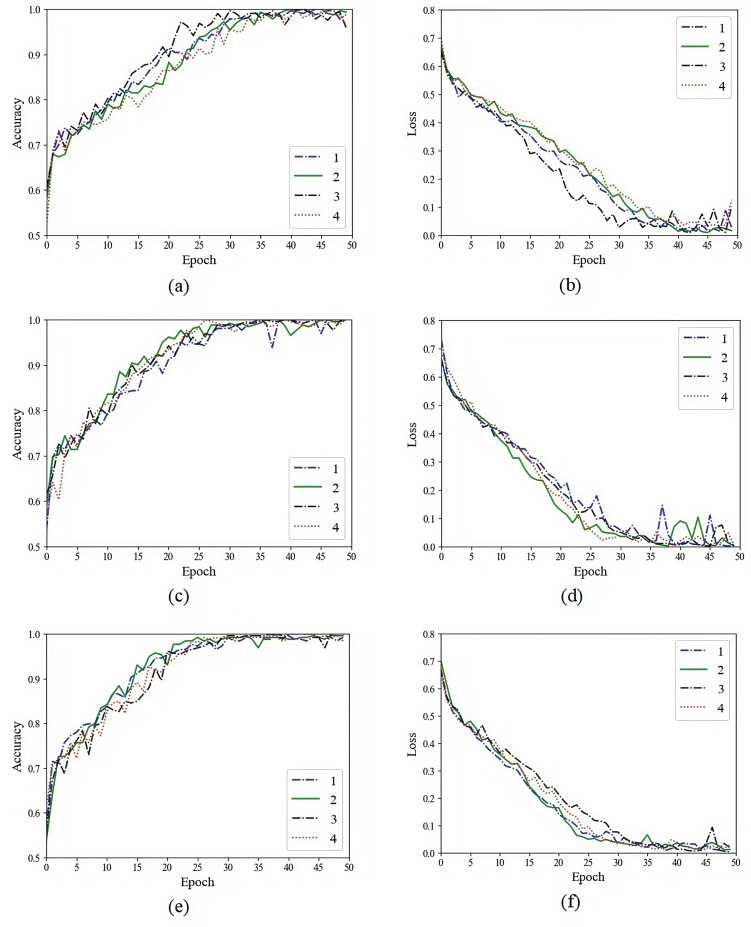
The glioma IDH1 classification training process of the improved U-Net model. (a) Multi-sequence training accuracy. (b) Multi-sequence training loss. (c) T1 training accuracy. (d) T1 training loss. (e) T2 training accuracy. (f) T2 training loss.

**Fig 12:**
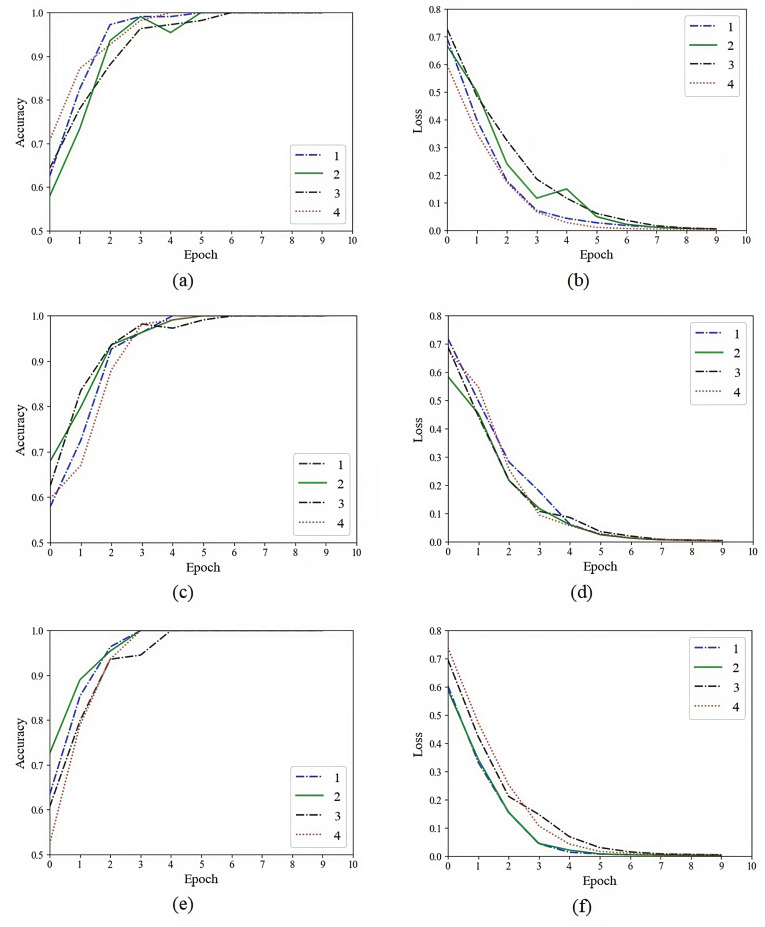
The pituitary tumor classification training process of the improved U-Net model. (a) Multi-sequence training accuracy. (b) Multi-sequence training loss. (c) T1 training accuracy. (d) T1 training loss. (e) T2 training accuracy. (f) T2 training loss.

Before training and testing, we randomly divided the whole data set of glioma or pituitary tumor images into a 80% training set and a 20% testing set. We input the feature sequences of the three modalities (multi-sequence, T1 and T2) extracted by the autoencoder model, the vanilla U-Net based model and the improved U-Net based model into the CRNN classifier for training, and recorded the training process. In order to ensure the reliability of the final test results, we repeated the above experimental process 4 times. Taking the feature sequence extracted by the improved U-Net based model as an example, the specific training process is shown in Figs 10–12. We trained the glioma model for 50 epochs and the pituitary tumor model for 10 epochs.

We recorded the average and variance of the test results. The performance of the autoencoder model, the vanilla U-Net based model and the improved U-Net based model on test sets is summarized in Tables 3–5. Our model achieved an average accuracy of 90.72% in the grading of glioma. In the classification of glioma IDH1 mutation status, an average accuracy of 94.35% were obtained. In the classification of pituitary tumors, an average accuracy of 94.64% were obtained. In addition, on the glioma data set from TCIA, our model achieved an average accuracy of 90.90% in the grading of glioma and an average accuracy of 93.94% in the classification of glioma IDH1 mutation status.

**Table 3:** Performance comparison of autoencoder, vanilla U-Net and improved U-Net in the grading of glioma.

	Autoencoder + CRNN (%)	Vanilla U-Net + CRNN (%)	Improved U-Net + CRNN (%)
multi-sequence	82.66 ± 2.88	84.68 ± 4.19	90.72 ± 1.33
T1	81.45 ± 1.85	81.46 ± 3.52	87.50 ± 1.76
T2	80.64 ± 2.28	83.88 ± 3.22	87.10 ± 1.98

**Table 4:** Performance comparison of autoencoder, vanilla U-Net and improved U-Net in the classification of the IDH1 status.

	Autoencoder + CRNN (%)	Vanilla U-Net + CRNN (%)	Improved U-Net + CRNN (%)
multi-sequence	85.49 ± 1.62	86.29 ± 5.76	94.35 ± 2.67
T1	83.87 ± 2.28	83.88 ± 3.72	89.52 ± 2.67
T2	82.26 ± 1.62	85.49 ± 3.23	88.71 ± 1.61

**Table 5:** Performance comparison of autoencoder, vanilla U-Net and improved U-Net in the classification of pituitary tumor.

	Autoencoder + CRNN (%)	Vanilla U-Net + CRNN (%)	Improved U-Net + CRNN (%)
multi-sequence	89.29 ± 2.53	84.82 ± 5.28	94.64 ± 3.18
T1	83.03 ± 2.96	82.14 ± 2.91	88.40 ± 5.86
T2	83.93 ± 5.36	83.93 ± 2.06	87.50 ± 5.36

**Fig 13:**
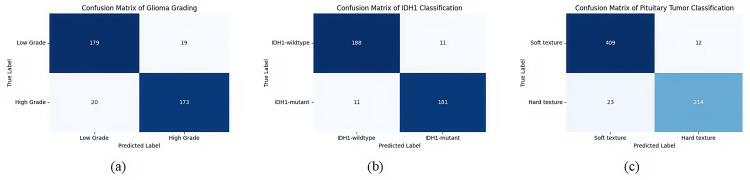
Performance of the improved U-Net in confusion matrix. (a) Confusion matrix of the improved U-Net in the grading of glioma. (b) Confusion matrix of the improved U-Net in the classification of glioma IDH1 mutation status. (c) Confusion matrix of the improved U-Net in the classification of pituitary tumors.

The performance of the improved U-Net in the grading of glioma, classification of glioma IDH1 mutation status and classification of pituitary tumors in confusion matrix is shown in Fig 13.

### 5.3. Comparisons with other models

Since deep learning has been widely used in the extraction and classification of tumor features and achieved good results, we validated our method by comparing the model used in this paper with some commonly used models [38,40–42] and some new models [19,43–48]. We implemented the experimental procedures in these references based on our dataset and trained the models for fine-tuning. The specific results are shown in Table 6.

**Table 6:** Comparisons of the grading or classification results using different methods.

Method	Feature extraction	Texture classification	Glioma grading accuracy (%)	Glioma IDH1 accuracy (%)	Pituitary tumor accuracy (%)
Simonyan et al. [40]	——	VGG	63.33	66.13	67.20
He et al. [41]	——	ResNet	72.52	72.58	76.23
Huang et al. [42]	——	DenseNet	73.54	75.81	79.25
Shi et al. [38]	——	CRNN	74.19	74.20	72.61
Deepak et al. [44]	——	GoogleNet + SVM	75.57	79.01	80.76
Yang et al. [43]	ROI	GoogleNet	79.58	82.24	83.33
Tang et al. [46]	——	GhostNetV2	81.83	90.85	91.03
This study	Autoencoder	CRNN	82.66	85.49	89.29
This study	Vanilla U-Net	CRNN	84.68	86.29	84.82
This study	Improved U-Net	CRNN	90.72	94.35	94.64
Choi et al. [19] (TCIA)	——	U-shaped + ResNet	–	78.80	–
Choi et al. [45] (TCIA)	——	FCNN + LSTM	–	91.70	–
Xu et al. [47] (TCIA)	——	TP-CA-Net	–	92.07	–
Zhao et al. [48] (TCIA)	——	CVT-RegNet	–	92.48	–
This study (TCIA)	Improved U-Net	CRNN	90.90	93.94	–

It can be seen from the Table 6 that comparing with other existing models, our proposed one has significant advantages. Particularly, comparing to the method that uses autoencoder or Vanilla U-Net as the feature extractor, the improved U-Net model that uses skip connections, Dense blocks and Residual blocks has better grading or classification performance. The use of Dense blocks and Residual blocks can enable the model to enhance feature reuse and avoid over-fitting while reducing the amount of parameters or deepening the network depth. Skip connection can make the model better integrate low-level and high-level features. These combined effects make the feature extraction model can extract more useful features and speed up the convergence speed. CRNN is a model for sequence data. It takes into account the relationship between slices and can help us extract features between slices to improve the accuracy of the entire model, so it is very suitable as a classifier for this experiment. The entire model uses image sequences instead of 2D (slice) classification methods. The effect of the multi-sequence fusion model is also significantly better than that of the single-sequence model.

In order to prove the statistical significance of the experiment, we used paired sample t-test to perform statistical test on the comparative experimental results. The specific data are shown in Table 7.

**Table 7:** Statistics of paired sample t-test.

Method	Feature extraction	Texture classification	Glioma grading (*P*)	Glioma IDH1 (*P*)	Pituitary tumor (*P*)
Simonyan et al. [40]	——	VGG	≤0.001	≤0.001	≤0.001
He et al. [41]	——	ResNet	≤0.001	≤0.001	≤0.001
Huang et al. [42]	——	DenseNet	≤0.001	≤0.001	≤0.001
Shi et al. [38]	——	CRNN	≤0.001	≤0.001	≤0.001
Deepak et al. [44]	——	GoogleNet^+^SVM	≤0.001	≤0.001	≤0.001
Yang et al. [43]	ROI	GoogleNet	≤0.001	≤0.001	0.006
Tang et al. [46]	——	GhostNetV2	≤0.001	≤0.001	≤0.001
This study	Auto-encoder	CRNN	0.003	0.002	0.014
This study	Vanilla U-Net	CRNN	0.022	0.031	0.022
This study	Improved U-Net	CRNN	≤0.001	≤0.001	≤0.001

**Fig 14:**
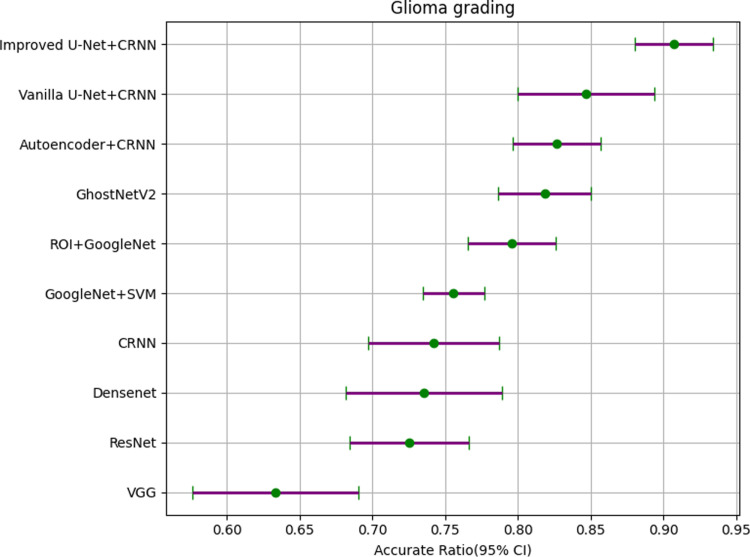
Forest plot of glioma grading.

**Fig 15:**
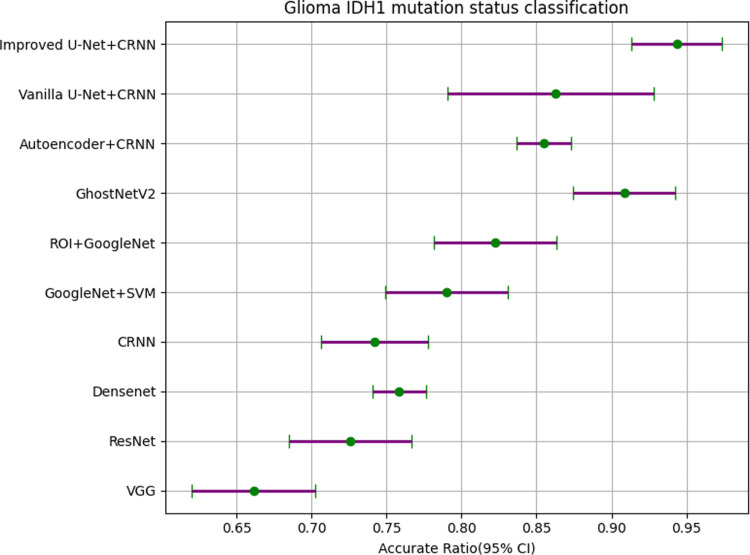
Forest plot of glioma IDH1 mutation status classification.

**Fig 16:**
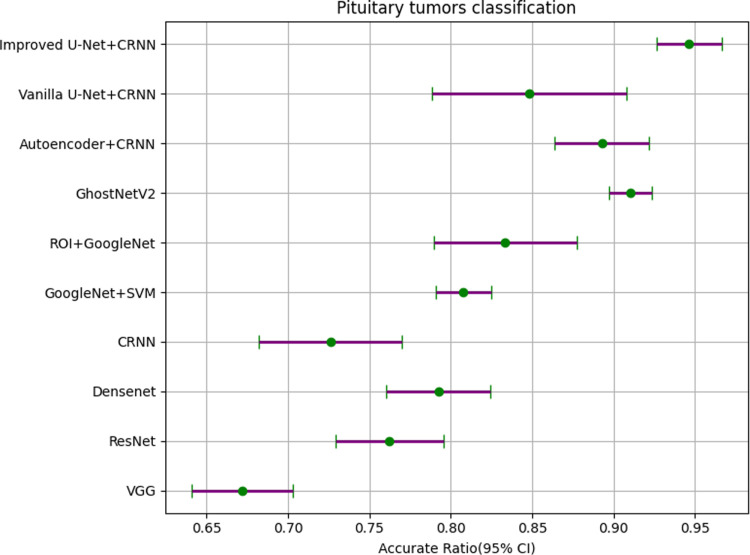
Forest plot of pituitary tumors classification.

It can be seen from Table 7 that the *P* values obtained by statistics on various models are all less than 0.05, which is statistically significant. The results are statistically significant. In order to express these results more intuitively, we made them into forest plots, as shown in Figs 14–16.

We show the details of the forest plots in Tables 8–10 to more clearly express the gap between different models.

**Table 8:** Forest plot data for glioma grading.

Feature extraction	Texture classification	Randomly Min	Randomly Max	Accurate Ratio (95% CI)
——	VGG	0.5806	0.7097	0.6333 (0.5763-0.6903)
——	ResNet	0.6774	0.7742	0.7252 (0.6842-0.7662)
——	DenseNet	0.6774	0.8065	0.7354 (0.6814-0.7894)
——	CRNN	0.7097	0.8065	0.7419 (0.6969-0.7869)
——	GoogleNet + SVM	0.7419	0.7742	0.7557 (0.7347-0.7767)
ROI	GoogleNet	0.7742	0.8387	0.7958 (0.7658-0.8258)
——	GhostNetV2	0.7912	0.8520	0.8183 (0.7863-0.8503)
Auto-encoder	CRNN	0.8065	0.8710	0.8266 (0.7966-0.8566)
Vanilla U-Net	CRNN	0.8065	0.9032	0.8468 (0.7998-0.8938)
Improved U-Net	CRNN	0.8710	0.9355	0.9072 (0.8802-0.9342)

**Table 9:** Forest plot data for glioma IDH1 mutation status classification.

Feature extraction	Texture classification	Randomly Min	Randomly Max	Accurate Ratio (95% CI)
——	VGG	0.6129	0.7097	0.6613 (0.6203-0.7023)
——	ResNet	0.6774	0.7742	0.7258 (0.6848-0.7668)
——	DenseNet	0.7419	0.7742	0.7581 (0.7401-0.7761)
——	CRNN	0.7097	0.7742	0.7420 (0.7060-0.7780)
——	GoogleNet + SVM	0.7419	0.8387	0.7901 (0.7491-0.8311)
ROI	GoogleNet	0.7742	0.8710	0.8224 (0.7814-0.8632)
——	GhostNetV2	0.8678	0.9463	0.9085 (0.8745-0.9425)
Auto-encoder	CRNN	0.8387	0.8710	0.8549 (0.8369-0.8729)
Vanilla U-Net	CRNN	0.8065	0.9355	0.8629 (0.7979-0.9279)
Improved U-Net	CRNN	0.9032	0.9677	0.9435 (0.9135-0.9735)

**Table 10:** Forest plot data for pituitary tumors classification.

Feature extraction	Texture classification	Randomly Min	Randomly Max	Accurate Ratio (95% CI)
——	VGG	0.6429	0.7143	0.6720 (0.6410-0.7030)
——	ResNet	0.7143	0.7857	0.7623 (0.7293-0.7953)
——	DenseNet	0.7500	0.8214	0.7925 (0.7605-0.8245)
——	CRNN	0.6786	0.7857	0.7261 (0.6821-0.7701)
——	GoogleNet + SVM	0.7857	0.8214	0.8076 (0.7906-0.8246)
ROI	GoogleNet	0.7857	0.8929	0.8333 (0.7893-0.8773)
——	GhostNetV2	0.8952	0.9241	0.9103 (0.8973-0.9233)
Auto-encoder	CRNN	0.8571	0.9286	0.8929 (0.8639-0.9219)
Vanilla U-Net	CRNN	0.7857	0.9286	0.8482 (0.7882-0.9082)
Improved U-Net	CRNN	0.9286	0.9643	0.9464 (0.9264-0.9664)

The experimental results suggest that our proposed improved U-Net based feature extraction model achieves considerably better performance in terms of grading or classification accuracy and convergence speed. Finally, the technology we propose is an end-to-end model. It relies only on medical images and does not need to have tumor segmentation in advance or manually extract a large number of features, which can save a lot of manpower, material resources and time.

## 6. Discussion

In this study, we construct brain tumor intelligent diagnosis based on brain tumor image sequences. This model consists of a feature extractor utilizing an improved U-Net and a classifier based on a CRNN. The feature extractor based on the improved U-Net is composed of an encoder based on a dense block and a decoder based on a residual block. The encoder performs feature extraction on the input glioma image sequence, and the decoder restores and outputs the feature sequence. The more similar the generated image is to the original image, the more representative the extracted features become. We input the feature sequence extracted by the feature extractor into the CRNN classifier for glioma grading, IDH1 classification and pituitary tumor classification. We constructed models for different sequences of brain tumor imaging, the T1 model, T2 model, and multi-modalities model.

The experimental results demonstrate that the method presented in this paper can enhance the accuracy of glioma grading, IDH1 classification and pituitary tumor classification As a result, this method holds promising applications in alleviating patient suffering, reducing economic burdens, and aid in clinical diagnosis.

Although our algorithm has achieved significant results in glioma grading, IDH1 classification, pituitary tumor classification, it also exhibits certain limitations. Firstly, the algorithm requires a substantial amount of MRI imaging data for training and validation, which may be constrained by data acquisition and processing in certain scenarios. Secondly, while we conducted model validation in our study, its generalization capability to other datasets or medical centers remains insufficiently validated and is contingent upon the stability of specific equipment and techniques. Additionally, the accuracy of brain tumors grading or classification remains a challenge, potentially leading to unstable model training or misleading outcomes. Moving forward, we will further research and refine the algorithm proposed in this paper to enhance its effectiveness and reliability in real- world clinical applications.

## 7. Conclusion

The preoperative grading of gliomas and the classification of IDH1 mutation status, and the preoperative classification of pituitary tumors are critical to the prognosis of patients and the formulation of surgical plans. But at present, it is impossible to diagnose these by visually observing MRI and methods such as biopsy are all invasive. This paper proposes a deep neural network model, based on improved U-Net and CRNN, for brain tumor intelligent diagnosis (grading of glioma, classification of glioma IDH1 mutation status and classification of pituitary tumor). This experiment finally achieved an average accuracy of 90.72% in the grading of gliomas. In the classification of glioma IDH1 mutation status, an average accuracy rate of 94.35% was obtained. In the classification of pituitary tumors, an average accuracy rate of 94.64% was obtained. In addition, on the glioma data set from TCIA, our model achieved a accuracy of 90.9% in the grading of glioma and a accuracy of 93.94% in the classification of glioma IDH1 mutation status. Experimental results suggest that our method has significant advantages, which include not only greatly improved accuracy on brain tumor grading or classification but also efficiency. This indicates that our method has great prospects in assisting clinicians to diagnose brain tumors and formulate treatment plans.
